# Current advances in haploid stem cells

**DOI:** 10.1007/s13238-019-0625-0

**Published:** 2019-04-19

**Authors:** Tongtong Cui, Zhikun Li, Qi Zhou, Wei Li

**Affiliations:** 1 State Key Laboratory of Stem Cell and Reproductive Biology, Institute of Zoology, Chinese Academy of Sciences, Beijing, 100101 China; 2 Institute for Stem Cell and Regeneration, Chinese Academy of Sciences, Beijing, 100101 China; 3 University of Chinese Academy of Sciences, Beijing, 100049 China

**Keywords:** haploidy, parthenogenetic, androgenetic, stem cells, diploidization, functional genomics, imprinting

## Abstract

Diploidy is the typical genomic mode in all mammals. Haploid stem cells are artificial cell lines experimentally derived *in vitro* in the form of different types of stem cells, which combine the characteristics of haploidy with a broad developmental potential and open the possibility to uncover biological mysteries at a genomic scale. To date, a multitude of haploid stem cell types from mouse, rat, monkey and humans have been derived, as more are in development. They have been applied in high-throughput genetic screens and mammalian assisted reproduction. Here, we review the generation, unique properties and broad applications of these remarkable cells.

## Introduction

Ploidy refers to the number of sets of chromosomes in a cell or organism, and is considered as a relatively stable cellular characteristic. Changes in ploidy may cause genomic instability, which was proved to promote cancer (Thompson and Compton, 2008; Potapova et al., 2013; Silk et al., 2013). Normally, diploid genomes are typical in most living animals as two homologous sets of chromosomes are existed per nucleus. As the dominant diploid phase is one of the major features of the life cycle in all mammals, haploid cells which contain only one set of chromosomes are generally restricted to gametes.

Evolutionarily speaking, the adaptive significance of diploidy over haploidy lies in two major perspectives. First, diploidy generates more variability or selective possibilities. Second, it is able to mask the deleterious recessive mutations (Paquin and Adams, 1983; Perrot et al., 1991). But when it comes to genetic analysis, these traits endow haploid cells with overwhelming advantages over diploid cells reversely. In haploid cells, loss-of-function mutations can be achieved in a single step, and phenotypes caused by recessive mutations can then be analyzed directly due to the lack of compensation for hemizygous gene mutations (Elling and Penninger, 2014; Wutz, 2014; Horii and Hatada, 2015; Li and Zhou, 2017). However, natural haploidy has not been reported in vertebrates, including mammals.

Thus for long, scientists have been trying to sought yeast-like systems for directly analyzing recessive and disease phenotypes, leading to alternative approaches like using unstable near-haploid cell cultures (Kotecki et al., 1999; Carette et al., 2009) or converting diploidy into haploidy by human-rodent cell fusions (Yan et al., 2000). Since 2009, with the constant optimization of derivation and culture techniques, haploid embryonic stem cell (haESC) lines from medaka fish, mouse, rat, monkey and even humans have been successfully established (Yi et al., 2009; Elling et al., 2011; Leeb and Wutz, 2011; Li et al., 2012; Yang et al., 2012, 2013; Li et al., 2014; Sagi et al., 2016). Most recently, the repertoire of haploid stem cells further expands to include haploid trophoblast stem cells (Cui et al., 2019; Peng et al., 2019), the extraembryonic counterpart of embryonic stem cells. In this review, we present an overview of existing haploid stem cells, as well as their strengths and limitations. We also explore ways in which these unique cells can deepen our understanding of mammalian development and reproductive approaches.

## Derivation of haploid mammalian embryos and haploid stem cells

Haploid embryos provide the source of haploid cell lines, which have long been experimentally produced by manipulation of gametes because spontaneous haploid embryos seldom occur naturally in mammals. There are two genetic origins of haploid animals, parthenogenetic (PG) and androgenetic (AG) haploid animals (Li and Zhou, 2017). PG embryos and AG embryos develop from only one of the gametes, the oocyte or the sperm, and therefore contain only the maternal genome or the paternal genome respectively. Parthenogenesis may occur naturally as in many insect species (Pamilo and Crozier, 1981; Beukeboom et al., 2007), or it may be induced experimentally. For parthenogenesis, there are two types. The first is the authentic parthenogenesis, in which chemical or electrical stimuli mimicking fertilization are applied directly to the oocyte and development is therefore initiated without fertilization. In the other circumstance called gynogenesis, the oocyte is stimulated by fertilization but the paternal chromatin is prevented from taking part in embryonic development (EDWARDS, 1957a, b, 1958; Wutz, 2014).

In 1975, Modliński pioneered in obtaining haploid mouse embryos by microsurgical removal of one pronuclei from fertilized eggs (Modliński, 1975), which is the first case in mammals after a success of such experiment on sea urchin eggs by Hiramoto in 1962 (Hiramoto, 1962). However, in the early trials to generate haploid mouse embryos, karyological investigations revealed that the obtained haploid embryos were almost all gynogenetic with an absence of androgenones. That phenomena might be explained by the fact that removal of the female pronucleus was more injurious to the ovum than removal of the male one, as the cell membrane in the region of female pronucleus is more delicate and sensitive to injury than in other parts due to abstraction of the second polar body at this site (Modliński, 1975; Tarkowski and Rossant, 1976). Of note, the parthenogenetic mouse blastocysts were reported to be a mixture of haploid and diploid cells, indicating that during the subsequent developmental process of the activated haploid oocytes, they may become diploid. Haploid cells could be detected up to the egg cylinder stage, though development of haploid embryos became progressively delayed (Tarkowski et al., 1970; Kaufman, 1978; Sagi and Benvenisty, 2017). Overall, these results suggested that it is possible to establish haploid pluripotent cells from the haploid embryos.

Experiments in the 1980s have pioneered the establishment of parthenogenetic haploid pluripotent cells. In 1983, Kaufman et al. attempted to establish pluripotent cell lines from parthenogenetic embryos, but chromosome analysis revealed that all of the four haploid-derived cell lines showed a diploid karyotype. Even though this technique provided a source of homozygous diploid cell lines of parthenogenetic origin (Kaufman et al., 1983), this study revealed a significant diploidization tendency of these haploid cells. Together with other works conducted in Drosophila and human showing that haploid cells were unstable and quickly diploidized (Debec, 1984; Kotecki et al., 1999), they raised the fundamental question about whether haploidy can fully support a stable growth as well as pluripotency in culture.

**Figure 1:**
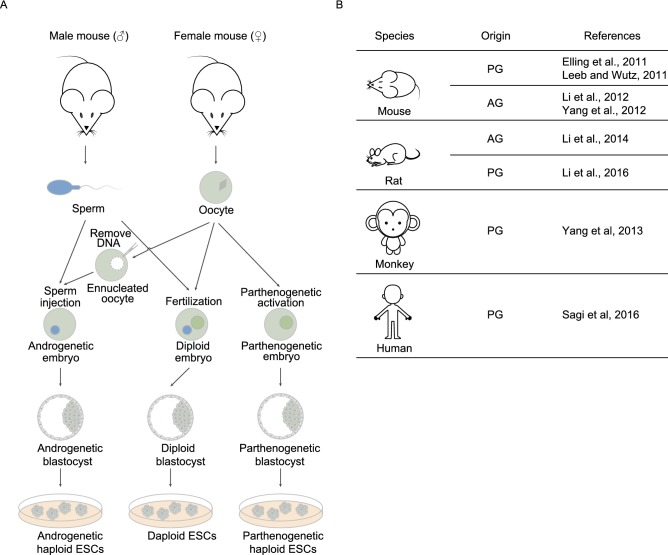
**Derivation of mouse haploid embryonic stem cells (haESCs)**. (A) Derivation strategies of parthenogenetic haESCs (phESCs) and androgenetic haESCs (ahESCs). Parthenogenetic haploid blastocysts are developed from artificially activated MII oocytes. Androgenetic embryos can be obtained by injecting sperm into the enucleated MII oocytes or removing the female pronucleus from fertilized oocytes. The resulting haploid blastocysts are subsequently cultured to develop haESC lines. (B) The haESC lines of different mammalian species have been generated

In 2009, Hong lab generated gynogenetic haploid medaka fish embryos and derived the first haESC lines, demonstrating that haploid genomes can be maintained in proliferating cells cultured *in vitro* (Yi et al., 2009). And the strong interest in deriving mammalian haESC lines revived. Two years later, two parallel studies reported the successful derivation of mouse parthenogenetic haESCs (phESCs) (Elling et al., 2011; Leeb and Wutz, 2011). Activation of unfertilized oocytes with strontium chloride or 5% ethanol together with subsequent *in vitro* culture of haploid embryos to the blastocyst stage enabled the establishment of haESC lines (Fig. 1A) (Elling et al., 2011; Leeb and Wutz, 2011). Notably, the application of flow cytometric cell sorting techniques allows for the selection of pure haploid cells with a G_1_ DNA content, which is a key progress. Meanwhile, advances in culture conditions also benefited the derivation and culture of haESCs (Bryja et al., 2006; Ying et al., 2008).

The established mouse phESCs exhibited a haploid karyotype, and largely maintain genome integrity. Sharing a similar transcriptional profile with diploid embryonic stem cells (ESCs), these haESCs express all classical pluripotency markers of diploid ESCs. Functionally, these haESCs can differentiate into lineages of all three germ layers in embryoid body (EB) formation assay. Importantly, these haESCs retain the *in vivo* differentiation potential as apparent coat color chimerism was observed after their being injected into diploid mouse blastocysts (Elling et al., 2011; Leeb and Wutz, 2011).

Hence, whether haESCs can function as haploid gametes to support fertilization and further development remained to be determined. We got the positive answer from androgenetic haESCs (ahESCs). In 2012, mouse ahESCs were established by injecting sperm into the enucleated metaphase II (MII) phase oocyte or removing the female pronucleus from fertilized oocytes (Fig. 1A) (Li et al., 2012; Yang et al., 2012). The ahESCs carry the paternal imprinting, though distinct from the sperm cells. Remarkably, these ahESCs can produce viable and fertile progenies after intracytoplasmic injection into mature oocytes. The production of fertile adult mice bearing haESC-carried genetic traits further shows that the genetic information in haESCs is functionally complete and stable, which significantly enhances the merits of haploid stem cells as a new tool to quickly generate genetic models (Li et al., 2012; Yang et al., 2012; Bai et al., 2016).

## Diversified haploid stem cells: from mouse to human

Subsequent trials in gamete manipulation have further yielded haESCs from other mammalian species including the rat and monkey (Fig. 1B) (Yang et al., 2013; Li et al., 2014). These cells with different origins possess a haploid karyotype, and share typical pluripotent stem cell characteristics, such as self-renewal capacity and a pluripotency-specific molecular signature. They are also approved amenable for genetic screening (Yang et al., 2013; Li et al., 2014; Li and Shuai, 2017). Notably, by fusing haESCs of two species, our lab reported the generation of mouse-rat allodiploid ESCs, which possess the pluripotency to differentiate into all three germ layers, and can serve as a powerful tool for identification of X inactivation-escaping genes as well as regulatory mechanisms between species (Li et al., 2016a).

Derivation of human haESCs had been hindered by the limited availability of human oocytes and spontaneous diploidization (Egli et al., 2011; Sagi and Benvenisty, 2017). As artificial activation of unfertilized MII human oocytes resulted in efficient development to the blastocyst stage and subsequent derivation of parthenogenetic ESCs (Kim et al., 2007; Revazova et al., 2007), characterization of these cell lines suggested that they were completely diploid (Paull et al., 2013; Sagi and Benvenisty, 2017). However, it was speculated that rare haploid cells might persist among the majority of diploid cells. The work of Sagi et al. led to the conclusion that human phESCs can be derived within successive rounds of haploid cell enrichment and expansion assisted by fluorescence activated cell sorting (FACS) (Sagi et al., 2016). Like other mammalian haESC lines, after being established, a sorting for the haploid population at every three to four passages is required to maintain the haploid stem cells (Leeb and Wutz, 2011; Li et al., 2012, 2014; Sagi et al., 2016). Notably, the EB generation assay and direct differentiation assays demonstrated that human haESCs can differentiate into various mature somatic cells while retaining a haploid genome. Haploid human neurons, cardiomyocytes and pancreatic cells were generated. In these haploid somatic cells, an X:autosomes dosage imbalance of 1:1 persisted into the differentiated state as haploid cells do not inactivate their single-copy X chromosome like in diploid female cells (Sagi et al., 2016).

However, it seemed more difficult to directly generate haploid somatic cells in other species as diploidization occurs very rapidly after differentiation of mouse, rat and monkey haESCs, which cannot be blocked by FACS-assisted purification (Elling et al., 2011; Leeb et al., 2012; Yang et al., 2013; Li et al., 2014; Sagi and Benvenisty, 2017). Based on a better understanding of the molecular mechanisms underlying diploidization, our lab recently showed that through ROCK inhibition, haploid somatic cellular fates of all three germ layers could be generated when haESCs were grown in defined mediums with different external growth-factor environments (He et al., 2017).

Besides the derivation of haESCs, extensive efforts have been made to devise robust protocols to generate other types of haploid pluripotent cells. Mouse epiblast stem cells (EpiSCs) are primed pluripotent stem cells, which could be derived from post-implantation embryos or via *in vitro* differentiation of ESCs (Brons et al., 2007; Tesar et al., 2007; Guo et al., 2009). As monkey and human haESCs were generated and shown to maintain haploidy in a putatively primed state (Yang et al., 2013; Sagi et al., 2016), it was also proved possible to generate both androgenetic and parthenogenetic mouse haploid EpiSCs (haEpiSCs) via *in vitro* differentiation from haESCs, which depends on the Activin/bFGF pathway to maintain self-renewal (Shuai et al., 2015). Subsequent work showed that ahESCs can develop into haploid neural stem cells (haNSCs) given appropriate signals, providing evidence to further prove that haESCs have the potential to undergo patterning events *in vitro* (Xu et al., 2017).

Trophoblast stem cells (TSCs) can be viewed as the extraembryonic developmental counterpart of ESCs. They originate from the outer trophectoderm layer, and are committed toward the trophoblast lineage from which ESCs are excluded (Tanaka et al., 1998; Tam and Rossant, 2003; Latos and Hemberger, 2016). As the culture condition of TSCs differs significantly from that of ESCs, it has remained elusive as whether haploid cell lines with a trophoblast lineage could be derived since the establishment of haESCs. A hint that this could be possible came from the early study showing that Gata6-induced extraembryonic cell fate might be compatible with a haploid genome (Leeb et al., 2012). Recently, we have reported the *de novo* generation of haploid trophoblast stem cells (haTSCs), which exhibit typical expression features of TSCs, possess the multipotency to differentiate into specialized trophoblast cell types and can chimerize developing placentas. Moreover, we showed that haTSCs can facilitate efficient genome-wide screening (Cui et al., 2019). Shuai lab also reported that overexpression of *Cdx2* together with deletion of *p53* can convert haESCs to haploid-induced TSCs (haiTSCs). By applying haiTSCs for high-throughput genetic screening, they found that *Htra1* is a blocker for spongiotrophoblast specification (Peng et al., 2019). The derivation of haTSCs and haiTSCs represents another interesting avenue that might help to explore fundamental biological roadmaps in the extraembryonic trophoblast lineage at a genomic scale.

## Being haploid: a fight against diploidization

Due to a strong tendency of spontaneous diploidization, it is difficult to maintain the haploid status over time without a frequent cytometric sorting of the G_1_ phase haploid cells at short intervals (Sagi and Benvenisty, 2017). Gaining more knowledge of principles governing the diploidization process will benefit advancing our experimental approaches to maintain haploidy in culture conditions. Early experiments with mixed cultures of haploid cells expressing different fluorescent proteins indicated that haploid cells do not become diploid via cell fusion, but via failed cytokinesis and/or endoreplication of the genome (Leeb et al., 2012).

Then it was proposed by Takahashi et al. that diploid conversion in haESCs might occur due to abnormal cell cycle regulation, i.e., by G_2_ arrest and abrupt insertion of an extra G_1_/S phase. They therefore tried to regulate the haploid cell cycle by adding inhibitors of Wee1 kinase. Experiments conducted in phESCs showed that acceleration of G_2_/M transition by means of Wee1 kinase inhibitors could prevent the spontaneous diploidization of haESCs and effectively maintain the haploid status for more than 4 weeks without FACS sorting conducted (Takahashi et al., 2014).

**Figure 2:**
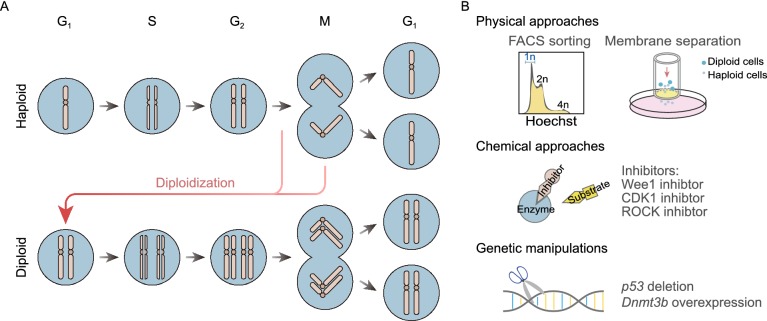
**Diploidization of haploid cells**. (A) Schematic showing that abnormal cell cycle regulation is the cause for diploidization in haploid cells. (B) Solutions for diploidization include physical approaches using fluorescence-activated cell sorting (FACS) and membranes with micrometer pores, chemical approaches via addition of kinase inhibitors and genetic manipulations in haploid cells

A recent study published by our lab showed that mitotic slippage, during which cells directly enter G_1_/S phase of the next cell cycle without division (Brito and Rieder, 2006), is a major cause of diploidization (Fig. 2A). After screening of a set of inhibitors related to cell cycle regulation, CDK1 and ROCK inhibitors were demonstrated to efficiently suppress diploidization during the culture and differentiation of haESCs (Fig. 2B) (He et al., 2017). Further experiments showed that supplementation with the ROCK inhibitor Y-27632 could facilitate the generation of haploid somatic cells and haTSCs (He et al., 2017; Cui et al., 2019).

There are also strategies focusing on manipulating the expression of particular genes to reduce diploidization. Olbrich et al. showed that *p53* deletion facilitates the maintenance of both human HAP1 cells (Carette et al., 2011b) and mouse haESCs by enabling the survival of genomically unstable cells. They also found that once diploidization occurs, diploid cells will rapidly overtake the culture owing to a better growth property (Olbrich et al., 2017). In another study, overexpression of *Dnmt3b*, the gene encodes the *de novo* DNA methyltransferase, was reported to mitigate the self-diploidization in ahESCs as certain G_2_/M-related genes were downregulated (Fig. 2B) (He et al., 2018).

Notably, based on the findings that haESCs are phenotypically smaller than diploid cells (Sagi et al., 2016), a new method for rapid purification of haploid ESCs from mixed cell populations with high viability was reported, which uses membranes with micrometer pores for force-free separation and allows haploid but not diploid cells to pass through (Fig. 2B). This method does not require the traditional periodic cell sorting and simplifies the culture procedures (Freimann and Wutz, 2017). Yet how efficiently can this approach facilitate the derivation of haploid cell lines remains to be explored.

## The haploid cell toolkit for functional genomics

A key goal in genetic analysis is to identify which genes contribute to specific biological phenotypes and diseases. As geneticists have long appreciated, a most effective way to probe genes influencing a phenotype of interest is via genetic screens. The hypothesis-driven, reverse genetic screens can test the effects of pre-defined gene mutations in cells, while forward genetic screens are hypothesis-free approaches that involve metagenesis, selection for the cells with a phenotype of interest, and then characterization of the causative mutation (Grimm, 2004; Shalem et al., 2015).

The genome-wide loss-of-function screen of recessive mutations is challenging in mammalian cells due to the diploid nature of their genomes, because it is time-consuming and rather difficult to generate genome-wide homozygous mutant libraries by standard genetic techniques. In this regard, haploid cells have remarkable advantages in forward genetic screens over diploid cells, as mutations in them are hemizygous and cellular phenotypes can be efficiently revealed (Elling and Penninger, 2014). Yeast cells can grow as haploids and have been used in a wide array of genome-wide phenotypic assays aimed toward an increased understanding of biological functions, response to stress and mechanisms of drug actions over the past decades (Forsburg, 2001; Giaever and Nislow, 2014). The establishment of mammalian haESCs proliferating with an intact haploid chromosome set has opened new and exciting avenues for high-throughput functional interrogation of the genome (Elling et al., 2011; Leeb and Wutz, 2011). Since then, the field of haploid screens has witnessed rapid development.

**Figure 3:**
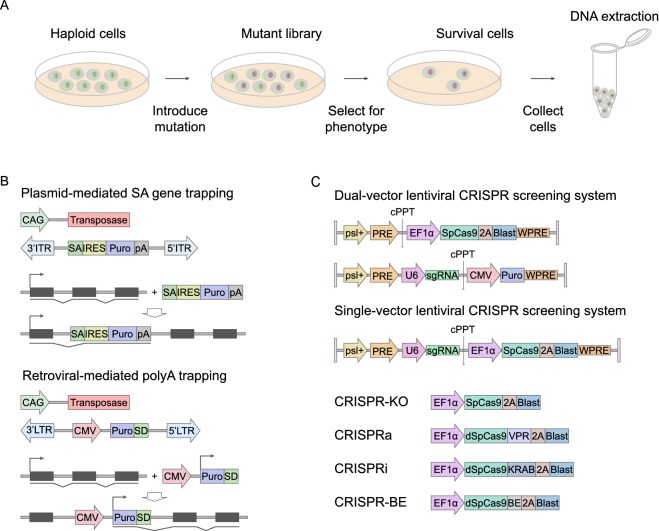
**Applying haploid stem cells for functional genomics**. (A) Forward genetic screens are powerful tools for the discovery and functional annotation of genetic elements. Three major steps are: mutagenesis to generate high-throughput mutant libraries, selection for the phenotype of interest, and mapping of mutations. (B) Two types of delivery systems used in gene trapping, the plasmid system and the retroviral system. (Top) Schematic diagram of splice acceptor (SA) gene trap, in which transposon elements were integrated in a plasmid vector. Splicing to upstream exons results in gene trap fusion transcript from which puromycin (puro) is transcribed by an endogenous promoter. CAG, a constitutive promoter; IRES, internal ribosome entry site; pA, poly A; ITR, inverted terminal repeat. (Bottom) Schematic diagram of ployA-trap, in which transposon elements were integrated in a retroviral vector. Insertion of a constitutive promoter-driven marker gene into introns results in a gene trap fusion transcript from which puromycin (puro) is terminated by an endogenous polyadenylation site. CMV, a constitutive promoter; SD, splice donor. LTR, long terminal repeat. (C) Schematic diagram of lentiviral expression vector for SpCas9 and sgRNA in a dual-vector form and single-vector form. psl+, Psi packaging singal; PRE, Rev response element; cPPT, central polypurine tract; EF1α, elongation factor 1α promoter; CMV, immediate-early cytomegalovirus enhancer-promoter; U6, RNA polymerase III U6 promoter; 2A, 2A self-cleavage peptide; Blast, blasticidin selection marker; Puro, puromycin selection marker; WPRE, post-transcriptional regulatory element

Like all genetic screens, haploid screens start with a mutagenesis step and are followed by the detection of a phenotype. Then the underlying genetic alteration is sought and correlated with a molecular function. In brief, there are three major steps: mutagenesis, selection and mapping of mutations (Fig. 3A). Of note, the validation of the hit target genes identified in secondary screens is also crucial (Elling and Penninger, 2014). Screening applications can be carried out in a wide range of formats using different molecular reagents and delivery vehicles (Fig. 3B and 3C). Usually, mutations in haploid cells are generated by gene trapping and nuclease-mediated gene knockout, including *piggyBac* and clustered regularly interspaced short palindromic repeats/CRISPR-associated nuclease (CRISPR/Cas) systems (Elling et al., 2011; Leeb and Wutz, 2011; Leeb et al., 2014; Monfort et al., 2015; He et al., 2017; Liu et al., 2017; Wang et al., 2018; Cui et al., 2019; Peng et al., 2019). Meanwhile, it was also reported to generate haESC libraries by chemical mutagens (Forment et al., 2016). Though chemical mutagenesis is easier to induce and results in a wider range of mutant alleles, the causal mutations in the selected clones are initially unknown, making identification of the causal mutations challenging. Different from that, insertional mutagenesis uses defined insertional sequences. Meanwhile, CRISPR sgRNAs can be synthesized to target specific sequences. Therefore, these two strategies have major advantages in the following mapping process as they are amenable to sequencing-based analysis (Elling and Penninger, 2014; Shalem et al., 2015).

Due to the vast number of cells and mutations, successful screens largely depend on the strong selection pressure as most haploid screens were based on lethality of cells with toxic agents or viruses and subsequent positive selection by outgrowth of resistant clones (Elling and Penninger, 2014). Cellular reporter systems were also reported (Leeb et al., 2014). To date, reported haploid cell lines have been applied for screens of cellular mechanisms including pathogen mechanisms, cellular pathways, gene essentiality, and targets of drug mechanisms (Table 1) (Wutz, 2014). Notably, the recent creation of Haplobank, a biobank of over 100,000 individual haESC lines targeting 16,970 genes with genetically barcoded, conditional and reversible mutations by genome-saturate mutagenesis, is a major breakthrough, which can be used in reverse and forward genetic screens for high-throughput genetic analysis (Elling et al., 2017).

**Table 1:** Genetic screens in haploid cell systems

Aim of the screen	Cell type	Strain	Genes identified	Reference
Mismatch repair pathway	haESCs	Mouse	*Msh2*	Leeb and Wutz, 2011
Ricin toxicity	haESCs	Mouse	*Gpr107*	Elling et al., 2011
Olaparib resistance	haESCs	Mouse	*Parp1*	Pettitt et al., 2013
Promotion of exit from ground state	haESCs	Mouse	*Zfp706, Pum1*	Leeb et al., 2014
X inactivation	haESCs	Mouse	*Spen*	Monfort et al., 2015
Mn^2+^ induced toxicity	haNSCLCs*	Mouse	*Park2*	He et al., 2017
A803467 toxicity	haNPCs^#^	Rhesus monkey	*B4GALT6*	Wang et al., 2018
Block against spongiotrophoblast specification	haiTSCs	Mouse	*Htra1*	Peng et al., 2019

*haploid neural progenitor cells; ^#^haploid neural stem-cell-like cells

Prior to the recent derivation of human haESCs (Sagi et al., 2016), a near-haploid leukemia cell line KBM-7 had been established from human tumors, which contains one copy of most chromosomes with the exception of Chromosome 8 and a portion of Chromosome 15 being disomic (Kotecki et al., 1999). And the attempt to reprogram KMB-7 into induce pluripotent stem cells (iPSCs) additionally yielded the haploid fibroblast-like cell line HAP1 (Carette et al., 2011b). These cells have been applied to genetic screenings for host genes required for action of toxins or viruses (Kotecki et al., 1999; Carette et al., 2009, 2011a, b; Baggen et al., 2016; Staring et al., 2017), and regulatory genes in biological pathways (Lebensohn et al., 2016). Now with haESCs introducing loss-of-function genetic screenings in human pluripotent cells, they provide new opportunities for functional genomics that would further advance our knowledge of human biology in health and diseases (Yilmaz et al., 2016).

## Replacing the gametes by haescs

**Figure 4:**
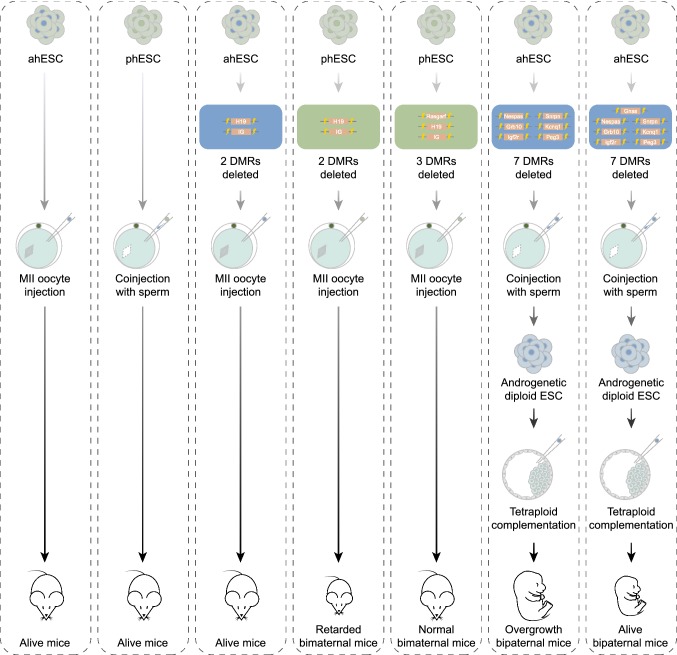
**Haploid embryonic stem cells (haESCs) can be used to replace gametes for the generation of alive mice**. Deletions of the specific imprinting regions in haESCs can facilitate to generate normally growing bimaternal mice and live bipaternal mice. DMRs, differentially methylated regions. ahESC, androgenetic haESC. phESC, parthenogenetic haESC

Upon the derivation of haESCs, it was unclear whether haESCs could be used as a substitute for one of the parental gametes. The important conclusion drawn from pioneering studies in mice and rats is that ahESCs have the ability to “fertilize” oocytes by intracytoplasmic injection and produce fertile adult mice with their genetic material being transmitted to the offspring (Fig. 4) (Li et al., 2012, 2014; Yang et al., 2012). Meanwhile, it was proved that phESCs could support embryo development via substituting the maternal genome (Fig. 4) (Wan et al., 2013). Compared with the mature gametes, haESCs are easily engineered and can proliferate indefinitely *in vitro*. Thus, besides opening a completely new avenue for generating genetically modified animals, they also provide a convenient platform to study effects of genetic and epigenetic issues on animal development, such as genomic imprinting.

Genomic imprinting is an epigenetic phenomenon that causes genes to be expressed in a parent-of-origin-specific manner. During fertilization, the zygote forms when a sperm carrying paternal imprints enters the oocyte with maternal imprints (Ferguson-Smith, 2011). Previous results showed that neither parthenogenetic nor androgenetic embryos could develop to term (Surani and Barton, 1983; McGrath and Solter, 1984; Surani et al., 1984, 1990). Examination of differentially methylated regions (DMRs) of imprinting genes showed that ahESCs might partially lose sperm-like methylation status during passaging (i.e., *H19* and *Gtl2*). Therefore, though live mice can be obtained by this approach, the developmental efficiency of ahESC-derived embryos is much lower than that of normal fertilized embryos (Li et al., 2012). The challenge was to improve the sperm-like features of ahESCs by optimizing their epigenetic makeup without compromising the genetic integrity and proliferative capacity. Subsequently, it was reported that with *H19-* and *IG-*DMRs being knocked out, ahESCs can stably retain the developmental potential and exhibit comparable “fertilizing” capacity as round spermatids (Fig. 4) (Zhong et al., 2015).

Methods to modify imprinting have been further developed. Previous work of our lab showed that *H19*- and *IG*-DMR deletions in phESCs enable the generation of vaible bimaternal mice with growth retardation after MII oocyte injection (Li et al., 2016b). In the following studies, we found that phESCs underwent global demethylation during *in vitro* culture: the demethylated DMRs of highly passaged phESCs mimicked the hypomethylated DMRs of PGCs (Li et al., 2018). After comparing the growth retarded bimaternal mice with the wild type control, we found a loss-of-imprint DMR in the somatic cells of bimaternal mice, *Rasgrf1*-DMR, which was also demethylated in the phESCs (Li et al., 2018). After deletions of *H19*-, *IG*- and *Rasgrf1*-DMRs in phESCs, we further derived the bimaternal mice with recovered growth curves. On the other hand, the PGC-like genome hypomethylation state was also found in highly passaged ahESCs. After deleting 7 DMRs (*Nespas*-, *Grb10*-, *Igf2r*-, *Snrpn*-, *Kcnq1*-, *Peg3*- and *Gnas*-DMRs) with CRISPR-Cas9 in ahESCs, they were co-injected into enucleated oocytes with sperm, and the bipaternal diploid blastocysts and androgenetic diploid ESCs (adESCs) were derived from the reconstructed embryos. Impressively, the diploid ESCs were able to produce full-term bipaternal mice after injecting into tetraploid blastocysts (Fig. 4). These results further proved that bipaternal reproduction barriers can also be crossed using haploid cells with specific imprinting regions being deleted (Li et al., 2018).

## Future perspectives

The mammalian haploid stem cells introduce new possibilities in a wide range of biological research fields and may offer unprecedented resolutions for genome exploration and reproductive approaches. The ahESCs can functionally take the place of sperms to produce live offspring after injection into the oocytes, which allows for the direct transmission of genomic modifications into the organism level without further time-consuming steps like conventional germline transmission. This is especially valuable for large animals and non-human primates. Therefore, the generation of monkey ahESCs and subsequent analysis of their capacity to take the place of sperms are worth trying for monkey genome engineering. Moreover, although we have gained some knowledge of principles governing diploidization, we are still woefully lacking in the molecular details and parameters that govern this phenomenon in haploid cells. Improving our understanding of this process is thus essential to the development of more stable culture conditions.

## Abbreviations

ahESC: androgenetic haESC
AG: androgenetic
CRISPR/Cas: clustered regularly interspaced short palindromic repeats/CRISPR-associated nuclease
DMR: differentially methylated region
EB: embryoid body
EpiSC: epiblast stem cell
ESC: embryonic stem cell
FACS: fluorescence activated cell sorting
haEpiSC: haploid EpiSC
haESC: haploid embryonic stem cell
haiTSC: haploid-induced trophoblast stem cell
haNSC: haploid neural stem cell
haTSC: haploid trophoblast stem cell
iPSC: induce pluripotent stem cell
MII: metaphase II
PG: parthenogenetic
phESC: parthenogenetic haESC
